# Supplementary material for: Biomarker discovery with quantum neural networks: a case-study in CTLA4-activation pathways
Source: BMC Bioinformatics. 2024 Apr 12;25:149. doi: 10.1186/s12859-024-05755-0 (PMC11265126; doi:10.1186/s12859-024-05755-0)
Supplement: Supplementary file 1 — Additional file 1. Supplementary Materials. [file 12859_2024_5755_MOESM1_ESM.pdf]

## Appendix A mRMR Criteria in The Context of Genetic Biomarker Discovery

An efficient and well-known method for genetic biomarker identification as feature selection problems is forward stage-wise search with *relevancy* and *redundancy* criteria [62]. The technique aims to simultaneously maximize the relevancy between a new feature $\boldsymbol{X}_i$ with target $\boldsymbol{Y}$

$$\mathrm{REL}(\boldsymbol{X}_i, \boldsymbol{Y}) := I(\boldsymbol{X}_i, \boldsymbol{Y}) \tag{12}$$

while minimizing the redundancy of the chosen set $\mathbb{S}$ [56]

$$\mathrm{RED}(\boldsymbol{X}_i|\mathbb{S}) := \sum_{\boldsymbol{X}_j \in \mathcal{S}} I(\boldsymbol{X}_i, \boldsymbol{X}_j). \tag{13}$$

Note that $I(\boldsymbol{X}_i, \boldsymbol{X}_j)$ is pair-wise mutual information computed from the feature set. The optimization problem is given as

$$\max_{\boldsymbol{X}_i \in \mathcal{X} \setminus \mathbb{S}} = \{\mathrm{REL}(\boldsymbol{X}_i) - \mathrm{RED}(\boldsymbol{X}_i|\mathbb{S})\}. \tag{14}$$

This problem is equivalent to minimizing the loss value:

$$\mathcal{L} = I(\boldsymbol{X}_i, \boldsymbol{Y}) - \lambda \sum_{\boldsymbol{X}_j \in \mathbb{S}} I(\boldsymbol{X}_i, \boldsymbol{X}_j), \tag{15}$$

where $\lambda = 1/|\mathbb{S}|$ is considered weights that scales the pairwise-mutual information between $\boldsymbol{X}_i$ and $\boldsymbol{X}_j$.

## Appendix B Quantum Unitary Transformation as Representations

We will present the quantum unitary transformation in QNNs as a representation.

**Definition 1** *A representation of a group $\mathcal{G}$ is a homomorphism $\Phi : \mathcal{G} \to GL(V)$ with $GL(V) := \{f \in End(V)|f$* ***is invetible***$\}$*. We have $End(V)$ as the endomorphism ring of (finite-dimensional) vector space $V$, or a set of linear mapping from $V$ to $V$.*

Let

$$\phi : \mathbb{Z}/n\mathbb{Z} \to \mathbb{C}^* \tag{16}$$

where $\mathbb{Z}/n\mathbb{Z}$ is the integer modulo $n$ and

$$\phi([m]) = e^{2\pi i m/n}.$$

The mapping induced by the direct sum $\oplus$:

$$\psi[m] = \begin{bmatrix} e^{\frac{2\pi m i}{n}} & 0 \\ 0 & e^{-\frac{2\pi m i}{n}} \end{bmatrix} \tag{17}$$

is equivalent to the representation $\mathbb{Z}/n\mathbb{Z} \rightarrow GL_2\mathbb{C}$:

$$\phi[m] = \begin{bmatrix} \cos(\frac{2\pi m}{n}) & -\sin(\frac{2\pi m}{n}) \\ \sin(\frac{2\pi m}{n}) & \cos(\frac{2\pi m}{n}) \end{bmatrix} \tag{18}$$

Thus, the quantum unitary transformations in **Equation** 4 and 6 are presented as the representation class $\psi[m]$ and $\phi[m]$, respectively. We note two main differences, which are (1) the flipped sign in $\psi[m]$ and $\boldsymbol{R_Z}$ and (2) the representation $\psi[m]$ and $\phi[m]$ intake rational values of $\frac{m}{n}$ while parameterized-rotation in **Equation** 4 and 6 intake real value. In the first difference, considering $\psi[-m]$ addresses the issue. Regarding the latter difference, taking a large value of $n$ will make the representation asymptotic in the quantum unitary transformations.

By generalizing the core of quantum AI as mathematical concepts in representation theory, we have introduced a mathematical approach to discovery in cancer.

## Appendix C Biological Meaning of Quantified Target Activation

We summarize the biological meaning of the quantified targets in **Table** C.

## Appendix D Supplemental Result of Quantum AI-driven Biomarkers

We show the inference report of the proposed model in **SuppFig** 1, 2, 3 and 4.

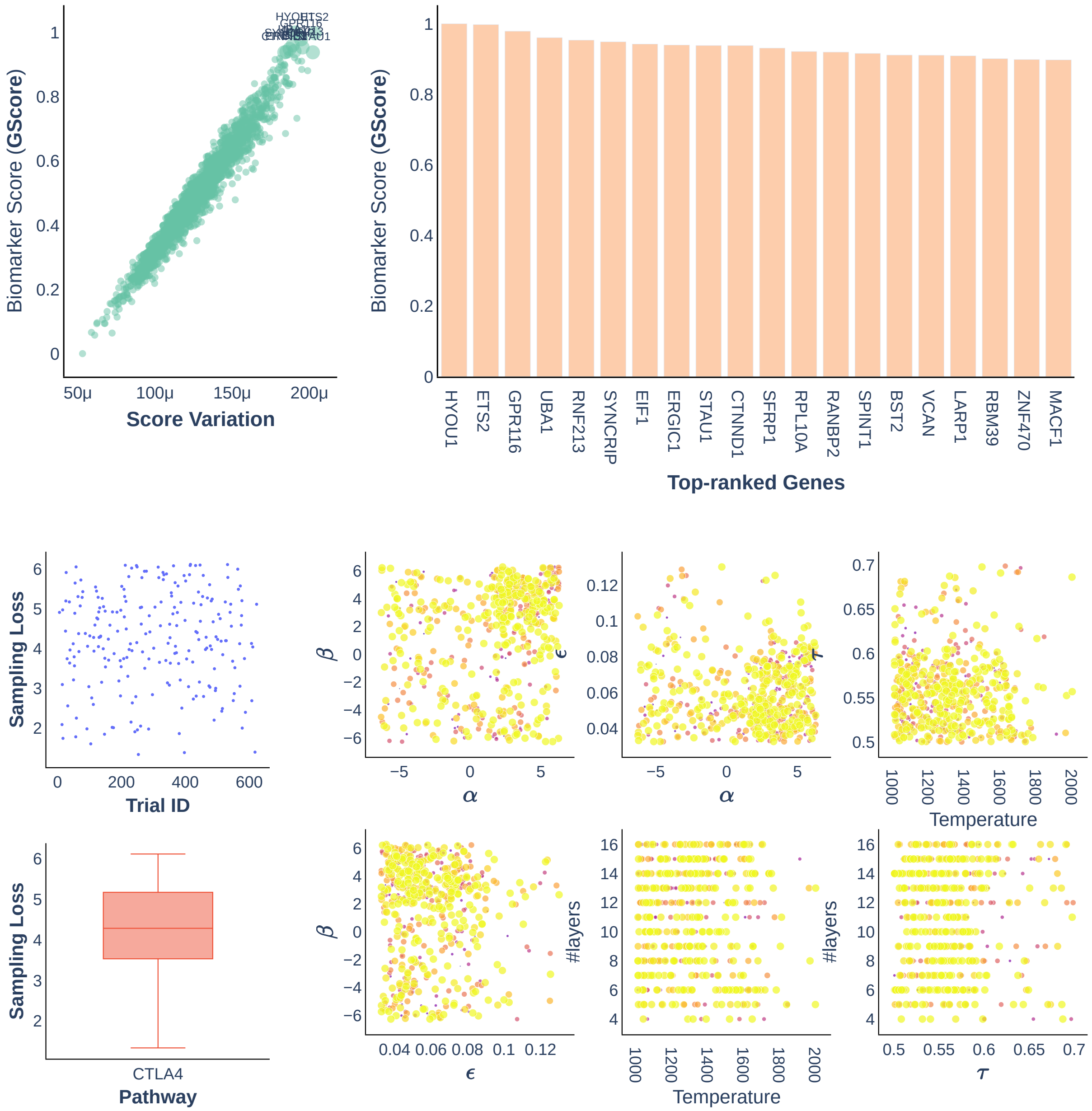


SuppFig. 1: **Experimental Report of Quantum AI-driven Genetic Biomarkers Discovered for *CTLA4* Pathway**. (Top) GSCORE© using top-50% samplers. The neural solutions are well-converged as the score variation is under $200\mu, \mu = 10^{-6}$. (Bottom) Convergence analysis of quantum sampler. The model configuration with a lower score is in darker color (purple), and the model configuration with a higher score is in brighter color (yellow).

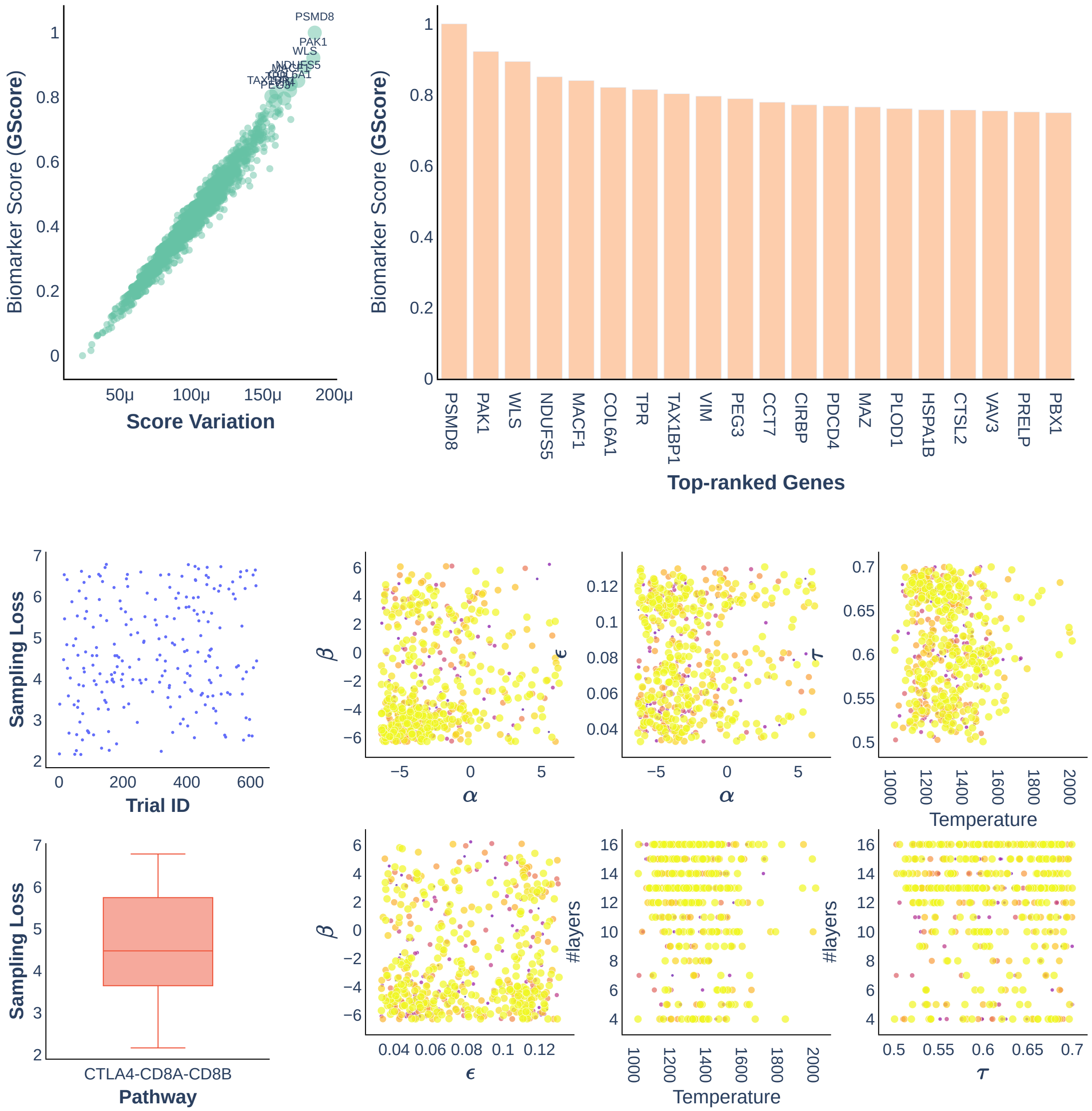


SuppFig. 2: **Experimental Report of Quantum AI-driven Genetic Biomarkers Discovered for *CTLA4-CD8A-CD8B* Pathway**. (Top) GSCORE© using top-50% samplers. The neural solutions are well-converged as the score variation is under $200\mu, \mu = 10^{-6}$. (Bottom) Convergence analysis of quantum sampler. The model configuration with a lower score is in darker color (purple), and the model configuration with a higher score is in brighter color (yellow).

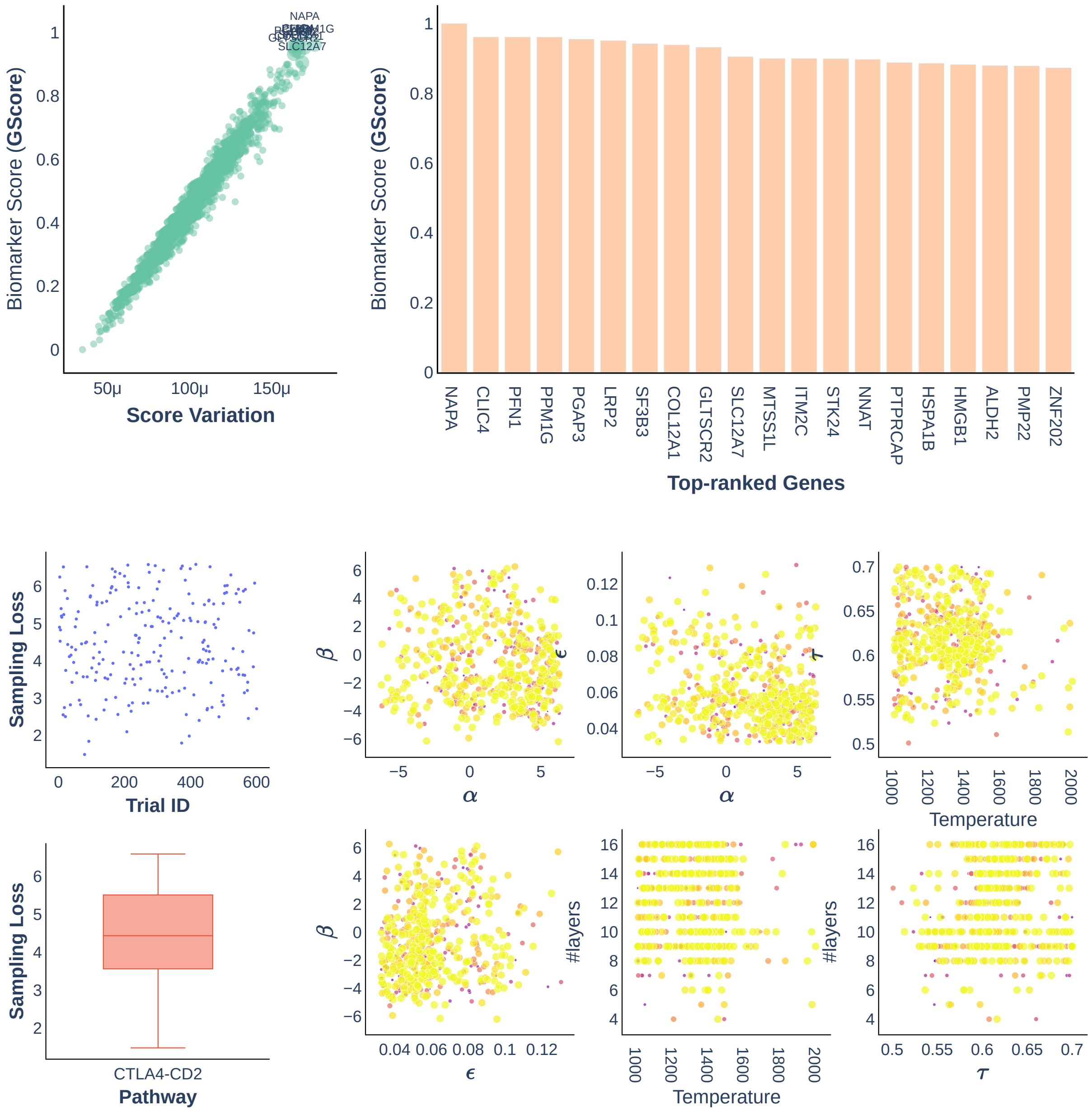


SuppFig. 3: **Experimental Report of Quantum AI-driven Genetic Biomarkers Discovered for *CTLA4-CD2* Pathway**. (Top) GSCORE© using top-50% samplers. The neural solutions are well-converged as the score variation is under $200\mu, \mu = 10^{-6}$. (Bottom) Convergence analysis of quantum sampler. The model configuration with a lower score is in darker color (purple), and the model configuration with a higher score is in brighter color (yellow).

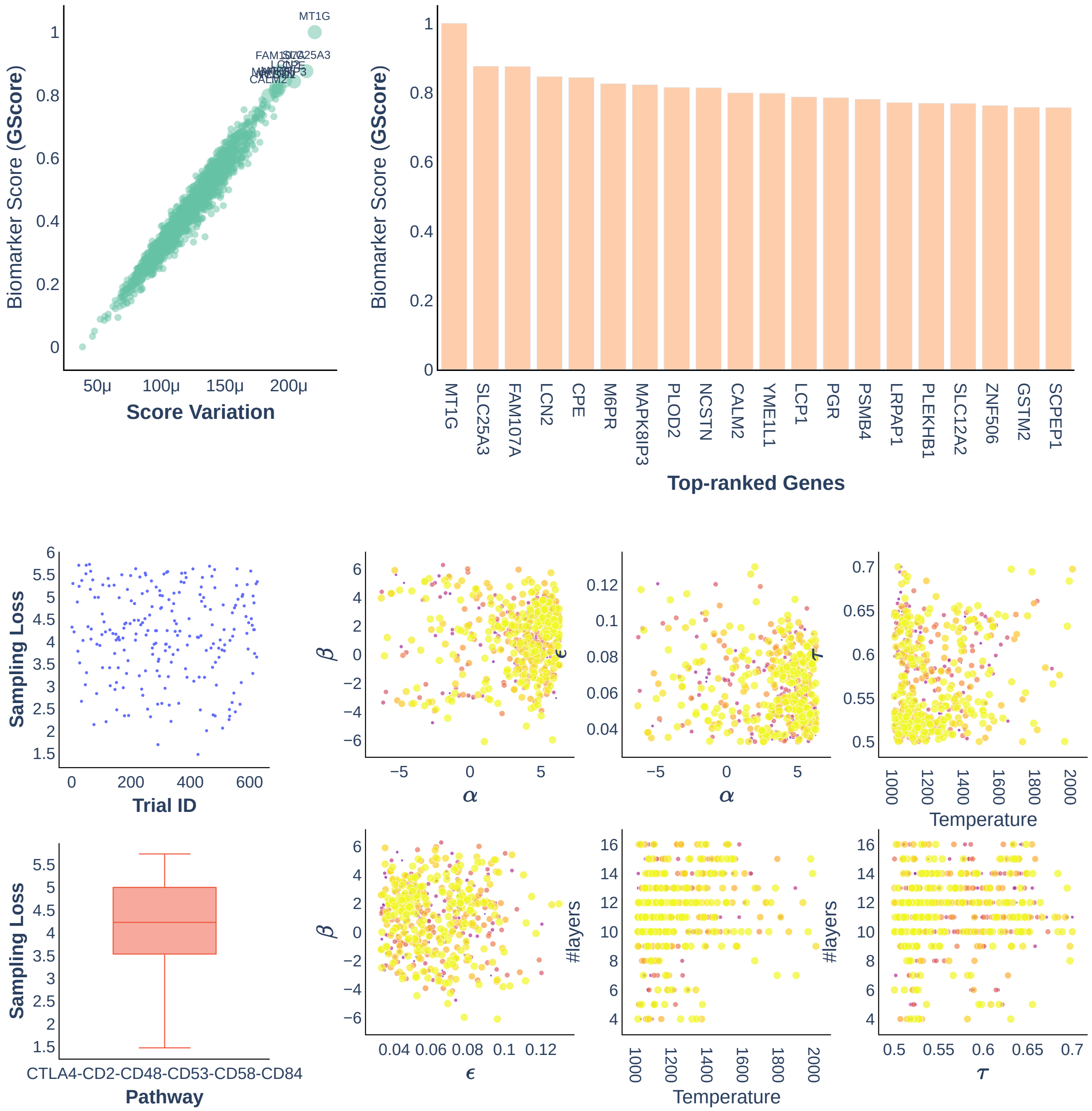


SuppFig. 4: **Experimental Report of Quantum AI-driven Genetic Biomarkers Discovered for *CTLA4-CD2-CD48-CD53-CD58-CD84* Pathway**. (Top) GSCORE© using top-50% samplers. The neural solutions are well-converged as the score variation is under $200\mu, \mu = 10^{-6}$. (Bottom) Convergence analysis of quantum sampler. The model configuration with a lower score is in darker color (purple), and the model configuration with a higher score is in brighter color (yellow).

SuppTab. 1: **Summary of Target Genes with Biological Meaning from [31].**

| Target Gene | Summary |
|---|---|
| *CTLA4* | This gene is a member of the immunoglobulin superfamily and encodes a protein that transmits an inhibitory signal to T cells. The protein contains a V domain, a transmembrane domain, and a cytoplasmic tail. Alternate transcriptional splice variants encoding different isoforms have been characterized. The membrane-bound isoform functions as a homodimer interconnected by a disulfide bond, while the soluble isoform functions as a monomer. Mutations in this gene have been associated with insulin-dependent diabetes mellitus, Graves disease, Hashimoto thyroiditis, celiac disease, systemic lupus erythematosus, thyroid-associated orbitopathy, and other autoimmune diseases. |
| *CD8A, CD8B* | The CD8 antigen is a cell surface glycoprotein found on most cytotoxic T lymphocytes that mediate efficient cell-cell interactions within the immune system. The CD8 antigen acts as a coreceptor with the T-cell receptor on the T lymphocyte to recognize antigens displayed by an antigen-presenting cell in class I MHC molecules. The coreceptor functions as either a homodimer composed of two alpha chains or a heterodimer composed of one alpha and one beta chain. Both alpha and beta chains share significant homology to immunoglobulin variable light chains. This gene encodes the CD8 alpha chain. Multiple transcript variants encoding different isoforms have been found for this gene. |
| *CD2* | A surface antigen of the human T-lymphocyte lineage is expressed on all peripheral blood T cells (summarized by Sewell et al., 1986 [PubMed 3490670]). It is one of the earliest T-cell markers on more than 95% of thymocytes; it is also found on some natural killer cells but not on B lymphocytes. Monoclonal antibodies directed against CD2 inhibit the formation of rosettes with sheep erythrocytes, indicating that CD2 is the erythrocyte receptor or is closely associated with it. |
| *CD48* | This gene encodes a CD2 subfamily of immunoglobulin-like receptors member, which includes SLAM (signaling lymphocyte activation molecules) proteins. The encoded protein is found on the surface of lymphocytes and other immune cells, dendritic cells, and endothelial cells and participates in activation and differentiation pathways in these cells. The encoded protein does not have a transmembrane domain but is held at the cell surface by a GPI anchor via a C-terminal domain, which may be cleaved to yield a soluble receptor form. Multiple transcript variants encoding different isoforms have been found for this gene. |
| *CD53* | The protein encoded by this gene is a member of the transmembrane four superfamily, also known as the tetraspanin family. Most of these members are cell-surface proteins that are characterized by the presence of four hydrophobic domains. The proteins mediate signal transduction events that play a role in the regulation of cell development, activation, growth, and motility. This encoded protein is a cell surface glycoprotein known to be complex with integrins. It contributes to the transduction of CD2-generated signals in T cells and natural killer cells and has been suggested to play a role in growth regulation. Familial deficiency of this gene has been linked to an immunodeficiency associated with recurrent infectious diseases caused by bacteria, fungi, and viruses. Alternative splicing results in multiple transcript variants. |
| *CD58* | This gene encodes a member of the immunoglobulin superfamily. The encoded protein is a ligand of the T lymphocyte CD2 protein and functions in the adhesion and activation of T lymphocytes. The protein is localized to the plasma membrane. Alternatively, spliced transcript variants have been described. |
| *CD84* | This gene encodes a membrane glycoprotein member of the signaling lymphocyte activation molecule (SLAM) family. This family forms a subset of the larger CD2 cell-surface receptor Ig superfamily. The encoded protein is a homophilic adhesion molecule expressed in numerous immune cell types and regulates receptor-mediated signaling in those cells. Alternate splicing results in multiple transcript variants. |

SuppTab. 2: **Literature Mining from *PubMed.gov* Library of Top-5 Discovered Genetic Biomarkers.** Markers with * are rarely known in clinical literature, mentioned in under 100 papers.

| Query | Paper Count |
|---|---|
| *SLC25A3** | 14 |
| *NDUFS5** | 8 |
| *PGAP3** | 20 |
| *WLS* | 224 |
| *LCN2* | 743 |
| *RNF213* | 180 |
| *PFN1* | 110 |
| *PAK1* | 353 |
| *FAM107A** | 21 |
| *PSMD8** | 6 |
| *MACF1** | 59 |
| *HYOU1** | 37 |
| *ETS2** | 92 |
| *PPM1G** | 19 |
| *NAPA* | 397 |
| *UBA1* | 200 |
| *CLIC4** | 51 |
| *GPR116** | 15 |
| *MT1G** | 41 |
